# Supplementary figures and images for: Switching between standard coral reef benthic monitoring protocols is complicated: proof of concept
Source: PeerJ. 2019 Dec 3;7:e8167. doi: 10.7717/peerj.8167 (PMC6896942; doi:10.7717/peerj.8167)

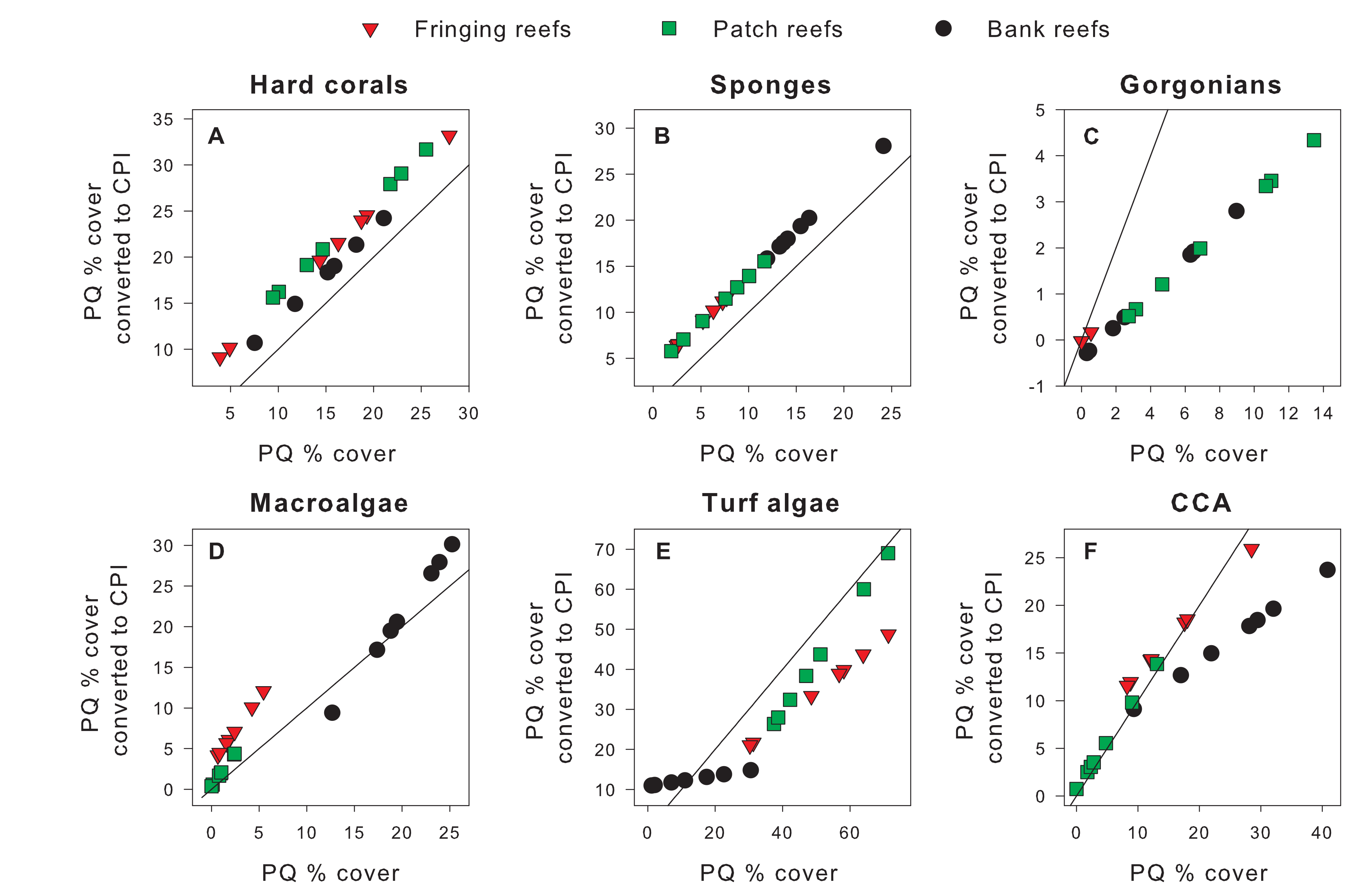

Supplement: Supplemental Information 1 — Benthic components shown are (A) hard corals, (B) sponges, (C) gorgonians, (D) macroalgae, (E) turf algae and (F) crustrose coralline algae. A solid line with an intercept equal to zero and a slope equal to one is shown in each panel for reference. Conversion formulae are given in Table 2. [file peerj-07-8167-s001.png]

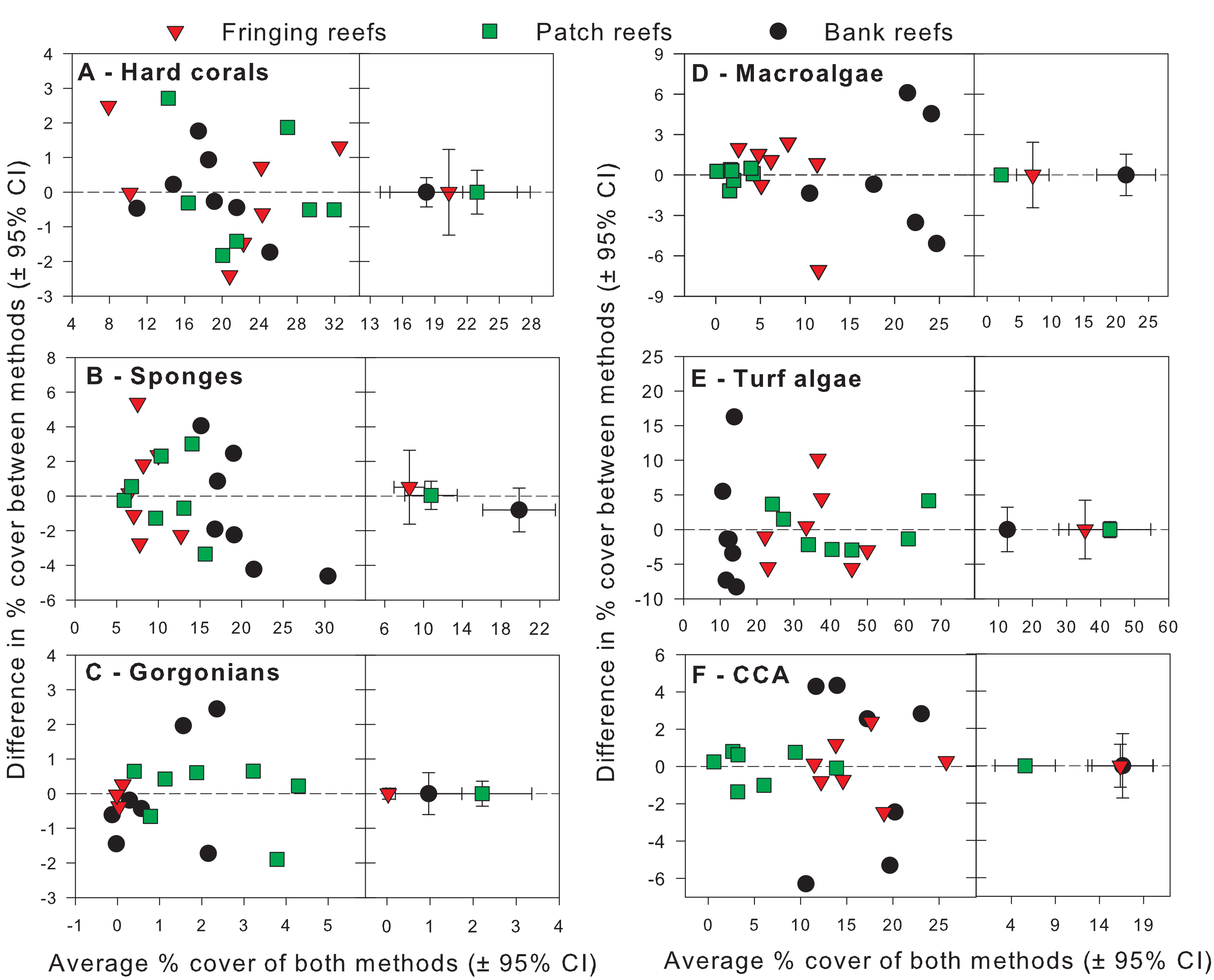

Supplement: Supplemental Information 2 — Benthic components shown are (A) hard corals, (B) sponges, (C) gorgonians, (D) macroalgae, (E) turf algae and (F) crustose coralline algae. Each panel shows site-specific values (left) and averages for each reef type (right) with corresponding 95% bootstrap confidence intervals (n = 7). Conversion formulae are given in Table 2. [file peerj-07-8167-s002.png]
