# Supplementary material for: Switching between standard coral reef benthic monitoring protocols is complicated: proof of concept
Source: PeerJ. 2019 Dec 3;7:e8167. doi: 10.7717/peerj.8167 (PMC6896942; doi:10.7717/peerj.8167)
Supplement: Supplemental Information 3 [file peerj-07-8167-s003.docx]

| Reef type | Site name | Site ID. | Lat/Long Coordinates | Depth (m) |
| --- | --- | --- | --- | --- |
| Bank | Maycocks Bank | A1 | N13 17 32.8 W59 39 47.5 | 18 |
| Bank | Speightstown Bank | A | N13 14 41.7 W59 39 08.1 | 18 |
| Bank | Allens Bank | B1 | N13 12 52.9 W59 38 53.9 | 21 |
| Bank | Holetown Bank | B | N13 11 00.9 W59 38 40.9 | 14.9 |
| Bank | Shark's Bank | D1 | N13 05 17.9 W59 38 00.4 | 18.9 |
| Bank | Hilton Bank | D | N13 04 10.9 W59 36 37.0 | 20.4 |
| Bank | Casuarina Bank | F | N13 03 18.8 W59 34 11.6 | 22.9 |
| Fringing | Sixmens | 1 | N13 16 17.7 W59 38 50.3 | 4 |
| Fringing | Mullins | 6 | N13 13 57.1 W59 38 43.8 | 6.7 |
| Fringing | Tropicana | 8 | N13 13 12.4 W59 38 38.2 | 5.5 |
| Fringing | Driftwood | 9 | N13 12 45.0 W59 38 32.6 | 4 |
| Fringing | Bachelor Hall | 11 | N13 11 58.6 W59 38 37.0 | 4.9 |
| Fringing | Sandy Lane | 14 | N13 10 19.8 W59 38 20.9 | 3.7 |
| Fringing | Fitts Village | 17 | N13 08 35.2 W59 38 21.7 | 3 |
| Patch | Carlisle Bay | 22 | N13 04 42.7 W59 36 52.9 | 4 |
| Patch | Asta | 24 | N13 04 28.3 W59 36 19.4 | 6.4 |
| Patch | Windsor Arms | 27 | N13 04 19.1 W59 35 46.8 | 9.8 |
| Patch | Blythwood | 29 | N13 04 09.2 W59 35 13.7 | 9.4 |
| Patch | Josef's | 31 | N13 03 52.1 W59 34 37.4 | 10.1 |
| Patch | Casuarina | 34 | N13 03 41.6 W59 33 38.7 | 4.9 |
| Patch | Union Villa | 36 | N13 03 45.6 W59 33 00.7 | 6.7 |
