# Supplementary material for: Switching between standard coral reef benthic monitoring protocols is complicated: proof of concept
Source: PeerJ. 2019 Dec 3;7:e8167. doi: 10.7717/peerj.8167 (PMC6896942; doi:10.7717/peerj.8167)
Supplement: Supplemental Information 4 — N/A—Information not available. [file peerj-07-8167-s004.docx]

| Method details | Study PQ | GCRMN PQ | BRMP CPI |
| --- | --- | --- | --- |
| No. of Transects per site | 11 | 5 | 10 |
| Transect length | 10 m | 30 m | 10 m |
| No. points per site | 1650 | 1875 | ~ 1300 |
| Transect placement | within 10 x 20 m plot | haphazard | within 10 x 20 m plot |
| No. photos per transect | 6 | 15 | - |
| Photo interval along transect | every 2 m | every 2 m | - |
| No. points per photo | 25 | 25 | - |
| No. photos per site | 66 | 75 | - |
| Photoquadrat size | 90 x 60 cm | 90 x 60 cm | - |
| Average time for *in situ*  data collection | 91 min per person per plot | N/A | 99 min per person per plot |
| Average time for *ex situ*  data processing^1^ | 265 min per person per plot | N/A | 133 min per person per plot |

^1^ Includes image processing time using (CPCe) software (Kholer & Gill 2006)
