# Supplementary material for: Switching between standard coral reef benthic monitoring protocols is complicated: proof of concept
Source: PeerJ. 2019 Dec 3;7:e8167. doi: 10.7717/peerj.8167 (PMC6896942; doi:10.7717/peerj.8167)
Supplement: Supplemental Information 5 — Percent cover are shown as means and standard deviations (n = 7 for each reef type). Original column shows the observed PQ values before conversion to chain point-intercept (CPI) values. The ANCOVA converted column shows the converted PQ values using the ANCOVA model outputs (see Table 2 for the corresponding conversion models). The MA regression converted column shows the converted PQ values using major axis regression. The latter was conducted by fitting individual models to each benthic component at each reef type (last column) using the “lmodel2” package (Legendre, 2018) in R. CCA, crustose coralline algae. [file peerj-07-8167-s005.docx]

| Benthic component | Reef type | Original | | ANCOVA converted | | MA regression converted | | MA regression models |
| --- | --- | --- | --- | --- | --- | --- | --- | --- |
|  |  | Mean | SD | Mean | SD | Mean | SD |  |
| Macroalgae | Fringing | 2.4 | 1.8 | 7.1 | 2.9 | 7.1 | 10 | CPI = -6.52 + 5.60 x PQ |
|  | Patch | 1.1 | 1.0 | 2.2 | 1.6 | 2.2 | 1.5 | CPI = 0.52 + 1.50 x PQ |
|  | Bank | 20.1 | 4.4 | 21.6 | 7.2 | 21.5 | 7.4 | CPI = -12.51 + 1.69 x PQ |
| Gorgonians | Fringing | 0.2 | 0.3 | 0.0 | 0.1 | 0.0 | 0.0 | CPI = 0.04 -0.09 x PQ |
|  | Patch | 7.5 | 4.2 | 2.2 | 1.5 | 2.2 | 1.7 | CPI = -0.75 + 0.39 x PQ |
|  | Bank | 3.8 | 3.4 | 1.0 | 1.2 | 1.0 | 0.5 | CPI = 0.41 + 0.15 x PQ |
| CCA | Fringing | 15.1 | 7.1 | 16.4 | 5.0 | 16.4 | 5.0 | CPI = 5.78 + 0.70 x PQ |
|  | Patch | 4.8 | 4.6 | 5.6 | 4.6 | 5.5 | 4.6 | CPI = 0.73 + 1.00 x PQ |
|  | Bank | 25.5 | 10.4 | 16.6 | 4.8 | 16.6 | 4.5 | CPI = 5.68 + 0.43 x PQ |
| Turf algae | Fringing | 51.5 | 15.8 | 35.3 | 10.6 | 35.5 | 10.5 | CPI = 1.28 + 0.66 x PQ |
|  | Patch | 50.3 | 13.0 | 42.5 | 16.3 | 42.7 | 16.4 | CPI = -20.95 + 1.27 x PQ |
|  | Bank | 13.1 | 10.9 | 12.5 | 1.4 | 12.6 | 0.5 | CPI = 13.21 -0.05 x PQ |
